# Altering retinol binding protein 4 levels in hepatitis C: Inflammation and steatosis matter

**DOI:** 10.1080/21505594.2020.1838742

**Published:** 2020-10-31

**Authors:** Ming-Ling Chang, Wei-Ting Chen, Jing-Hong Hu, Shiang-Chi Chen, Po-Wen Gu, Rong-Nan Chien

**Affiliations:** a Division of Hepatology, Department of Gastroenterology and Hepatology, Chang Gung Memorial Hospital, Taoyuan, Taiwan; b Department of Medicine, College of Medicine, Chang Gung University, Taoyuan, Taiwan; c Department of Internal Medicine, Chang Gung Memorial Hospital, Yunlin, Taiwan; d Department of Nursing, Taipei Medical University, Taipei, Taiwan; e Department of Medical Laboratory, Chang Gung Memorial Hospital, Taoyuan, Taiwan; f Division of Biotechnology, Graduate Institute of Biomedical Sciences, College of Medicine, Chang Gung University, Taoyuan, Taiwan

**Keywords:** HCV, RBP4, steatosis, inflammation, insulin resistance, HOMA-IR

## Abstract

Both hepatitis C virus (HCV) infection and retinol-binding protein 4 (RBP4) might contribute to insulin resistance (IR), how RBP4 links to IR in HCV infection remain elusive. A joint study of a prospective cohort of 842 chronically HCV-infected (CHC) patients (with 842 controls) and a line of HCV core transgenic mice was conducted. Of 842 patients, 771 had completed anti-HCV therapy and 667 had sustained virological responses (SVRs). Compared with controls, CHC patients had lower RBP4 levels. At baseline, age (95% CI β: −0.87~−0.317), BMI (0.516~2.036), triglycerides (0.03~0.127), neutrophil-to-lymphocyte ratio (NLR) (1.561~7.327), and estimated glomerular filtration rate (eGFR) (−0.342~−0.149) levels were associated with RBP4 levels in CHC patients. At 24-week post-therapy, male sex (0.652~8.129), BMI (0.199~1.254), triglycerides (0.039~0.088), uric acid (0.599~3.067), eGFR (−0.247 ~−0.14) levels, and fibrosis-4 (−3.602~−0.039) scores were associated with RBP4 levels in SVR patients; compared with baseline, except genotype 3 HCV-infected patients, SVR patients had increased RBP4 levels, which were comparable with controls, while no HOMA-IR index alteration was noted after SVR. The HCV core transgenic mice exhibited nonobese hepatic steatosis, had higher hepatic RBP4 expression, higher serum levels of RBP4 and triglycerides, but comparable HOMA-IR levels than non-transgenic littermates. In conclusion, steatosis, sex, age, uric acid, NLR, and FIB-4 levels were associated with HCV-related RBP4 levels; BMI, triglycerides, and eGFR levels were associated with non-HCV-related RBP4 levels. Reversal of low RBP4 levels after SVR was evident in non-genotype 3 HCV-infected patients. Steatosis and inflammation linked with metabolic alteration other than IR, determined RBP4 levels in HCV-infected patients.

## Introduction

**Figure 1.**
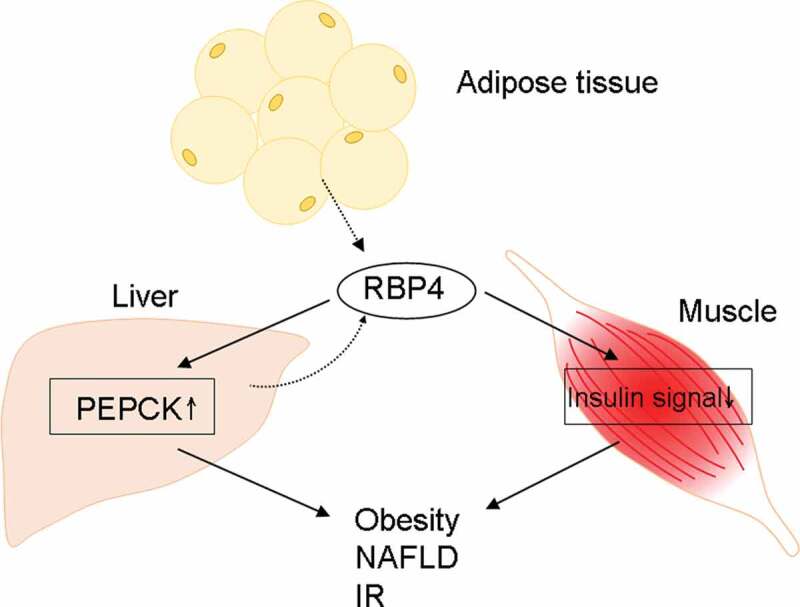
**A schematic flow chart of the pathways affected by RBP4**. PEPCK: phosphoenolpyruvate carboxykinase; NAFLD: nonalcoholic fatty liver disease. Solid line: promotion; dashed line: secretion. IR: insulin resistance

Retinol-binding protein 4 (RBP4) is a 21-kDa protein that facilitates the transport of retinol through the circulation to peripheral tissues [1]. Hepatocytes are the primary producers of RBP4. Under lean conditions, adipocytes express about one-fifth as much RBP4 mRNA as a hepatocyte [2], while this expression increases substantially in obesity [3]. RBP4 was found to induce hepatic expression of the gluconeogenic enzyme phosphoenolpyruvate carboxykinase and impaired insulin signaling in the muscle of mice [4]. It was therefore suggested to connect obesity-associated comorbidities, especially insulin resistance (IR), and certain components of the metabolic syndrome such as nonalcoholic fatty liver disease (NAFLD), in either retinol-dependent or retinol-independent way, with RBP4 [4,5]. A flow chart of RBP4-associated pathways had been shown in Figure 1. However, clinical data regarding the links among RBP4, IR, and NAFLD are conflicting [6–9]. The inconclusiveness mainly arose from heterogeneous hepatic and renal functions in these studies, as liver and kidney are the main source and catabolism organs for RBP4, respectively [2,10]. For example, plasma RBP4 levels tended to decrease concomitantly with increased necroinflammatory activity, NAFLD activity score, and fibrosis score in NAFLD patients [9,11,12], but increase during chronic kidney disease [10].

Hepatitis C virus (HCV), a human pathogen responsible for acute and chronic liver disease, has variants classified into eight genotypes [13] and chronically infects an estimated 71.1 million individuals worldwide [14]. HCV is now thought to cause metabolic alterations in addition to being a simple hepatic viral infection, as it affects the insulin signaling and much of its life cycle is closely associated with lipid metabolism [15]. Several lines of evidences indicate the involvement of HCV in retinoid pathway: HCV core protein enhances retinoid X receptor-α-dependent transcriptional activity [16], and antagonizes all-trans retinoic acid to stimulate cell growth via epigenetic down-regulation of retinoic acid receptor-β2 [17]; HCV NS3/4A protein disrupts retinoic acid-inducible gene I signaling pathways to halt the defense against HCV [18]. However, the relationship between HCV infection and RBP4 remained elusive. Although one study of nondiabetic, nonobese patients with genotype 1 (G1) chronic hepatitis C (CHC) showed that serum RBP4 level was positively linked to viral steatosis and CHC patients had higher RBP4 levels than the controls [19], other CHC studies either failed to link RBP4 positively with steatosis [11,20], or demonstrated a lower RBP4 level than the controls [11]. Given the inconclusive relationship between HCV infection and RBP4 levels, how RBP4 affects metabolism including IR in CHC patients remains even more unclear.

Accordingly, we sought to elucidate the precise relationships among HCV infection, RBP4, and IR by conducting a prospective CHC cohort study analyzing the profiles before and after anti-HCV therapy, as comparing the pre- and post-therapy profiles within the same patients with sustained virological responses (SVRs) has provided an excellent opportunity to eliminate the individual bias affecting RBP4 levels [9–12]. RBP4 levels from normal controls were used to verify the completeness of the RBP4 alteration reversal after viral clearance. In parallel, the impact of HCV core on RBP4 expression was assessed by using the tetracycline (tet)-off conditional HCV core transgenic mice with nonobese hepatic steatosis and hypolipidemia [21–24], both phenotypes mimicked HCV-associated metabolic alterations in the human [15]. For both human and animal studies, immunohistochemical (IHC) studies were performed to survey the hepatic RBP4 expression.

## Materials and methods

### Patients

The study group comprised subjects ≥18 years with CHC, which was defined as detectable HCV RNA for ≥24 weeks. The controls were identified by the absence of HCV infection. Subjects with bacteria, human immunodeficiency virus or hepatitis B virus infection, hemochromatosis, primary biliary cholangitis, primary sclerosing cholangitis, autoimmune hepatitis or malignancy, and recipients of solid organ transplants were excluded.

### Human study

**Figure 2.**
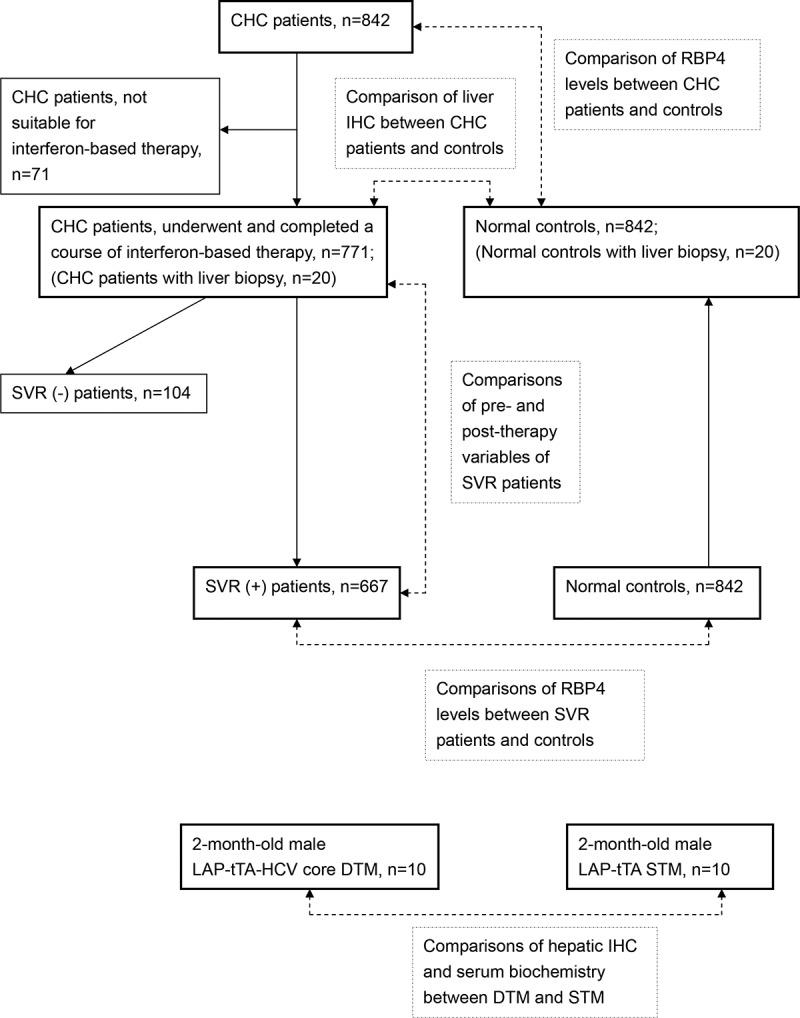
**A schematic flow chart of the enrolled subjects**. CHC, patients with chronic hepatitis C virus infection; SVR, sustained virological response: IHC, immunohistochemistry studies; RBP4, retinol-binding protein 4; LAP-tTA-HCV core DTM, liver activator protein promoter-tetracycline transactivator-HCV core double transgenic mice; LAP-tTA STM, liver activator protein promoter-tetracycline transactivator single transgenic mice

A schematic flow chart for all the enrolled patients is shown in Figure 2. The study group was composed of 842 CHC patients, recruited consecutively at a tertiary referral center between July 2010 and January 2018. The control group was composed of 842 sex- and age-matched subjects, enrolled from the health management center in the hospital between July 2010 and January 2018. For all included CHC patients, several baseline factors were evaluated, including sex, age, body mass index (BMI), HCV RNA and genotype, presence of hepatic steatosis and cirrhosis, estimated glomerular filtration rate (eGFR), total cholesterol (TC), triglycerides (TG), high-density lipoprotein-cholesterol (HDL-C), homeostasis model assessment-estimated insulin resistance (HOMA-IR) [fasting insulin (μU/mL) × fasting glucose (mmol/L)/22.5] index, fibrosis-4 (FIB-4) [(Age (years) × aspartate transaminase (U/L)/(Platelets (10^9^/L) × (√(ALT (U/L))] index, platelet count, uric acid, alanine aminotransferase (ALT), thyroid-stimulating hormone (TSH), RBP4 (R&D Systems, Minneapolis, MN) levels [25] and interferon λ3 (IFNL3) single-nucleotide polymorphism (SNP) genotypes [26,27]. The presence of fatty liver or cirrhosis was screened by abdominal sonography and confirmed by Fibroscan. Of 842 CHC patients, 771 had completed a course of anti-HCV therapy with weight-based pegylated interferon-α-2b and ribavirin for either 24 or 48 weeks [26,27]. Both pegylated interferon and ribavirin pose antiviral and immunomodulatory activities [28]. For the patients who had completed anti-HCV therapy, the aforementioned factors were evaluated 2 weeks before therapy, and 24 weeks after the end of therapy. IR was defined as a HOMA-IR index ≥2.5. An SVR was defined as undetectable levels of HCV RNA 24 weeks after the completion of therapy. For controls, the serum RBP4 was assessed by using fasting serum. Liver biopsy was performed in CHC patients before anti-HCV therapy (n = 20). Control liver samples were acquired from the livers of sex- and age-matched participants taken from the tissue bank of the hospital (n = 20). IHC studies of RBP4 (Phoenix Pharmaceuticals, CA, USA) were performed using paraffinized liver samples according to the manufacturers’ protocols. Protein expression intensity was determined as described previously [29].

### HCV core transgenic mice study

FVB/N mice in which expression of the HCV core gene (from G1b HCV) was suppressible by tetracycline were raised as previously described [21–24]. The liver protein was extracted from 2-month-old male liver activator protein (LAP) promoter-tetracycline transactivator (tTA)-HCV core double transgenic mice (DTM) (n = 10) and 2-month-old male LAP-tTA single transgenic mice (STM) (n = 10) as described previously [24]. The STM served as the controls for the DTM. Liver biopsy samples were either frozen or fixed in freshly prepared 4% paraformaldehyde for IHC examination. The cells were permeabilized with 0.1% Triton-100; incubated with HCV core (Anogen/YES Biotech Laboratories Ltd, Mississauga, Canada) or RBP4 antibodies [Proteintech Group, Inc., IL, USA, 1:100 dilution (low antibody concentration to avoid over staining)]; washed, and then incubated with secondary antibody. Fat vesicles in the livers were identified by Oil Red O staining of frozen sections according to the manufacturer’s protocol (BioGenex, Fremont, CA). The mice serum RBP4 and insulin levels were measured using commercially available enzyme-linked immunosorbent assay kits (R&D Systems, Minneapolis, MN), according to manufacturer instructions. The assays for fasting serum glucose, TG, TC, and ALT levels (Vitros DT60 II Chemistry System; Johnson & Johnson, Rochester, NY) were adopted by using fasting tail blood according to the manufacturer’s protocol. The protein expression intensity was determined as described previously [28]. The flow chart of the mice is also shown in Figure 2.

### Statistics

All statistical analyses were performed using either Statistical Product and Service Solutions (SPSS ver. 21.0, SPSS Inc., Chicago, IL, USA) or MedCalc (MedCalc ver. 12.4, MedCalc Software Corp., ME, USA) software. The continuous variables were summarized as the means ± standard deviation (SD), and categorical variables as frequencies and percentages. To compare different variables in different groups, continuous variables were analyzed using Student’s t-tests, whereas categorical variables were analyzed using chi-squared or Fisher’s exact tests as appropriate. Multivariate linear regression models were used to assess the relationship between dependent and independent factors by adjusted for all independent variables with *p* value <0.1 in univariate analyses. Paired t-tests were used to compare variables prior to and 24 weeks after completion of anti-HCV therapy within individuals. Statistical significance was defined at the 5% level based on two-tailed tests of the null hypothesis.

### Institutional review board

Informed consent in writing was obtained from each patient, and the study protocol conformed to the ethical guidelines of the 1975 Declaration of Helsinki and was approved by the Chang Gung Memorial Hospital institutional review board. The animal care and use committee at the hospital approved the use of animals in this study.

## Results

### Baseline characteristics

The baseline characteristics of the CHC patients are shown in Table 1. Of 842 CHC patients, with a mean age of 55.46 years, 462 (54.9%) were males, 771 had completed a course of interferon-based therapy, and 667 had SVRs (Figure 2). Compared with 667 SVR patients, the 104 non-SVR patients had higher rates of G1 HCV infection and liver cirrhosis, and higher levels of HCV RNA and HOMA-IR and FIB-4 scores, and lower rates of G2 and G3 HCV infections, and platelet counts. The baseline RBP4 levels between the SVR and non-SVR patients were comparable (Table 1). Compared with normal controls, the CHC patients had lower baseline RBP4 levels (33.8±28.6 vs. 46.2±35.6 μg/mL, *p* < 0.001).

**Table 1.** Baseline characteristics of CHC patients

	All (*n* = 842)	SVR (+) (*n* = 667)	SVR (−) (*n* = 104)	*p* Value*
Male gender, *n* (%)	462 (54.9)	386 (57.8)	56 (53.8)	0.477
Age (years)	55.46±12.40	53.49±11.74	55.26±10.94	0.162
BMI (kg/m^2^)	24.82 ±3.79	24.85±3.60	25.66±4.11	0.05
HCV genotype				
genotype 1, *n* (%)	456 (54.2)	312 (46.8)	83 (79.8)	<0.001
genotype 2, *n* (%)	306 (36.3)	307 (46.0)	16 (15.4)	<0.001
genotype 3, *n* (%)	23 (2.73)	20 (2.99)	(0)	<0.001
Log HCV RNA (logIU/mL)	5.97±1.09	5.88±1.15	6.46±0.72	<0.001
ALT (U/L)	96.31±99.26	101.06±104.03	83.76±76.56	0.130
eGFR (mL/min/1.73 m^2^)	99.41±37.23	102.84±34.06	105.74±44.68	0.58
TG (mg/dL)	103.47±53.49	102.86±49.67	112.04±63.63	0.120
TC (mg/dL)	170.21±33.35	172.12±31.95	173.05±28.84	0.792
HDL-C (mg/dL)	47.60±14.00	47.74±13.88	47.65±14.30	0.955
HOMA-IR	3.23 ±5.32	2.96±4.38	4.25±5.26	0.035
Uric acid (mg/dL)	5.92 ±1.56	5.97±1.56	5.82±1.48	0.405
NLR	1.69±0.93	1.63±0.89	1.65±0.68	0.841
Platelet (10^3^/uL)	174.44±63.33	181.23±57.87	155.91±57.53	<0.001
Liver cirrhosis, *n* (%)	190 (22.6)	121 (18.1)	37 (35.6)	<0.001
Fatty liver, *n* (%)	403 (47.9)	332 (50.1)	47 (45.2)	0.249
Fibrosis-4 score	3.43±3.43	2.89±3.01	3.81±3.86	0.542
TSH (mU/L)	1.97±2.57	1.82±1.34	1.91±1.05	0.034
RBP4 (μg/mL)	33.8±28.6	32.9±17.5	40.0±61.0	0.324
IFNL3-rs12979860 CC genotype, *n* (%)	714 (84.8)	580 (87.0)	86 (82.7)	0.397

CHC, chronic hepatitis C virus infection; SVR, sustained virological response; BMI, body mass index; HCV, hepatitis C virus; RNA, ribonucleic acid; ALT, alanine transaminase; eGFR, estimated glomerular filtration rate; TG, triglycerides; TC, total cholesterol; HDL-C, high-density lipoprotein cholesterol; HOMA-IR, homeostatic model assessment for insulin resistance; NLR, neutrophil-to-lymphocyte ratio; TSH, thyroid-stimulating hormone; RBP4, retinol-binding protein 4; IFNL3, interferon-λ3. *P values between SVR (+) and SVR (−) patients.

### Baseline associations of RPB4 levels in CHC patients

Among 842 CHC patients, the baseline levels of BMI, TG and NLR were positively associated with RBP4 levels and the levels of age and eGFR were negatively associated with RBP4 levels (Table 2).

**Table 2.** Associations of RBP4 levels in CHC patients at baseline

Baseline factors	Univariate analyses		Multivariate analyses	
	95% CI of β (β)	*p* Value	95% CI of β (β)	*p* Value
Male, yes	2.75 to 12.93 (7.84)	0.003	−1.997 to 8.527 (3.265)	0.223
Age (years)	−0.532 to −0.088 (−0.31)	0.006	−0.87 to −0.317 (−0.593)	<0.001
BMI (kg/m^2^)	0.167 to 1.508 (0.837)	0.014	0.516 to 2.036 (1.276)	0.001
HCV genotype	−6.69 to 1.95 (−2.37)	0.282		
Log HCV RNA (logIU/mL)	−0.804 to 4.161 (1.678)	0.185		
ALT (U/L)	−0.043 to 0.004 (−0.002)	0.108		
eGFR (mL/min/1.73 m^2^)	−0.315 to −0.156 (−0.236)	<0.001	−0.342 to −0.149 (−0.246)	<0.001
TG (mg/dL)	0.048to 0.144 (0.096)	<0.001	0.03 to 0.127 (0.078)	0.002
TC (mg/dL)	−0.049 to 0.107 (0.029)	0.471		
HDL-C (mg/dL)	−0.243 to 0.245 (−0.054)	0.576		
HOMA-IR	−0.549 to 0.663 (0.057)	0.853		
Uric acid (mg/dL)	2.115 to 5.412 (3.764)	<0.001	−1.095 to 2.726 (0.815)	0.402
NLR	1.706 to 7.789 (4.748)	0.002	1.561 to 7.327 (4.464)	0.003
Platelet (10^3^/µL)	0.003 to 0.084 (0.044)	0.037	−0.06 to 0.042 (−0.009)	0.732
Liver cirrhosis, yes	−10.83 to 2.39 (−4.22)	0.211		
Fatty liver, yes	−3.12 to 7.53 (2.20)	0.416		
Fibrosis-4 score	−2.02 to −0.55 (−1.285)	0.001	−1.875 to 0.005 (−0.935)	0.051
TSH (mU/L)	−1.81 to 2.35 (0.267)	0.801		
IFNL3-rs12979860 CC genotype, yes	−7.7 to 28.5 (10.42)	0.259		

RBP4, retinol-binding protein 4; CHC, chronic hepatitis C virus infection; CI, confidence interval; BMI, body mass index; HCV, hepatitis C virus; RNA, ribonucleic acid; ALT, alanine transaminase; eGFR, estimated glomerular filtration rate; TG, triglycerides; TC, total cholesterol; HOMA-IR, homeostatic model assessment for insulin resistance; NLR, neutrophil-to-lymphocyte ratio; IFNL3, interferon-λ3.

### Post-therapy associations of RBP4 in SVR patients

Among 667 SVR patients, at 24 weeks post-therapy, male sex, levels of BMI, TG, and uric acid were positively associated with RBP4 levels and the levels of eGFR and FIB-4 scores were negatively associated with RBP4 levels (Table 3).

**Table 3.** Associations of RBP4 levels in SVR patients at 24 weeks post-therapy

24-Week post-therapy factors	Univariate analyses		Multivariate analyses	
	95% CI of β (β)	*p* Value	95% CI of β (β)	*p* Value
Male, yes	4.564 to 11.494 (8.029)	<0.001	0.652 to 8.129 (4.391)	0.022
Age, (years)	−0.245 to 0.074 (−0.085)	0.294		
BMI (kg/m^2^)	0.199 to 1.242 (0.721)	0.007	0.199 to 1.254 (0.726)	0.007
ALT (U/L)	−0.035 to 0.224 (0.094)	0.153		
eGFR (mL/min/1.73 m^2^)	−0.22 to −0.111 (−0.165)	<0.001	−0.247 to −0.14 (−0.191)	<0.001
TG (mg/dL)	0.026 to 0.058 (0.042)	<0.001	0.039 to 0.088 (0.064)	<0.001
TC (mg/dL)	0.032 to 0.127 (0.079)	0.001	−0.066 to 0.03 (−0.018)	0.468
HDL-C (mg/dL)	−0.264 to 0.007 (−0.128)	0.064	−0.029 to 0.287 (0.129)	0.011
HOMA-IR	−0.579 to 0.679 (0.05)	0.875		
Uric acid (mg/dL)	2.78 to 4.93 (3.85)	<0.001	0.599 to 3.067 (1.833)	0.004
NLR	−0.248 to 4.434 (2.093)	0.08	−0.076 to 3.147 (2.035)	0.059
Platelet (10^3^/uL)	0.019 to 0.082 (0.005)	0.002	−0.043 to 0.04 (−0.001)	0.946
Liver cirrhosis, yes	−13.18 to −3.9 (−8.55)	<0.001	−9.525 to 0.256 (−4.635)	0.063
Fatty liver, yes	−0.469 to 6.805 (3.168)	0.088	−4.252 to 2.343 (0.955)	0.569
Fibrosis-4 score	−3.13 to −0.487 (−1.808)	0.007	−3.602 to −0.039 (−1.82)	0.045
TSH (mU/L)	0.082 to 2.486 (1.284)	0.036	−0.497 to 1.517 (0.51)	0.32
IFNL3-rs12979860 CC genotype, yes	−11.96 to 11.08 (−0.438)	0.94		

RBP4, retinol-binding protein 4; SVR, sustained virological response; CI, confidence interval; BMI, body mass index; ALT, alanine transaminase; eGFR, estimated glomerular filtration rate; TG, triglycerides; TC, total cholesterol; HOMA-IR, homeostatic model assessment for insulin resistance; NLR, neutrophil-to-lymphocyte ratio; IFNL3, interferon-λ3.

### Longitudinal alterations of RBP4 and HOMA-IR levels in SVR patients

Compared with baseline levels, only SVR but not non-SVR patients had increased RBP4 levels at 24 weeks post-therapy; however, among the SVR patients, no significant alteration of RBP4 levels was noted in those infected with G3 HCV (Table 4). Compared with normal control, the SVR patients had similar post-therapy RBP4 levels (45.23±16.34 vs. 46.2±35.6 μg/mL, *p* = 0.735). On the other hand, compared with baseline, no significant alteration of HOMA-IR levels was noted, regardless of SVR and HCV genotype (Table 4).

**Table 4.** Comparisons between pre-therapy and 24-week post-therapy RBP4 levels and HOMA-IR of CHC patients

		HCV genotype	Pre-therapy	24-week post-therapy	*p* Value (pre- vs. post-therapy)
	RBP4 (μg/mL)				
SVR (−)			33.58 ± 18.62	47.51 ± 67.84	0.082
SVR (+)			32.76 ± 17.90	45.27 ± 16.42	<0.001
		Genotype 1	33.68 ± 20.45	46.46 ± 16.31	<0.001
		Genotype 2	31.18 ± 12.82	43.32 ± 15.89	<0.001
		Genotype 3	44.26 ± 36.46	50.53 ± 24.45	0.11
		Other genotypes	31.02 ± 12.60	49.11 ± 16.38	0.001
	HOMA-IR				
SVR (−)			4.22 ± 5.32	4.49 ± 7.07	0.512
SVR (+)			2.95 ± 4.42	2.69 ± 2.74	0.07
		Genotype 1	3.12 ± 0.58	2.81 ± 3.52	0.172
		Genotype 2	2.76 ± 2.59	2.60 ± 1.76	0.399
		Genotype 3	2.68 ± 2.70	2.54 ± 2.35	0.685
		Other genotypes	3.34 ± 3.41	2.41 ± 1.58	0.331

CHC, chronic hepatitis C virus infection; SVR, sustained virological response; HOMA-IR, homeostatic model assessment for insulin resistance.

### Human hepatic RBP4 expression

**Figure 3.**
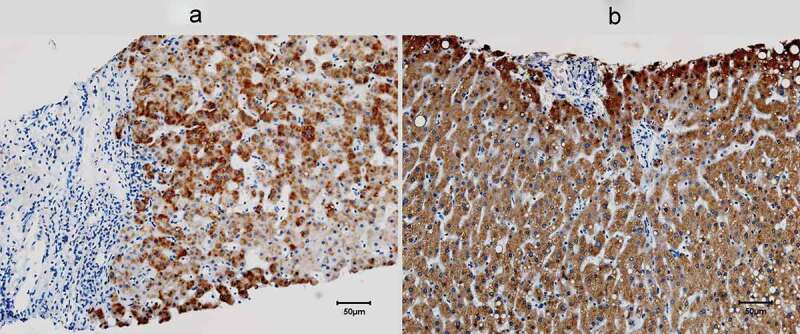
**Hepatic expression of RBP4 of a representative 63-year-old male CHC patient** (a) **and a 63-year-old male control** (b). RBP4 was stained in brown, and most RBP4-positive cells were hepatocytes. The areas of hepatic inflammatory infiltration and fibrosis exhibited negligible RBP4 expression

Compared with normal controls, the pre-therapy livers from the CHC patients exhibited more inflammatory infiltration and fibrosis but lower levels of RBP4 expression (68.23±22.56 vs. 95.88 ±12.05%, *p* = 0.012) (Figure 3).

### HCV core transgenic mice serum biochemistry

The LAP-tTA-HCV core DTM had higher levels of ALT, TG, and RBP4, and lower levels of TC than that of tTA STM. The HOMA-IR levels were comparable between the DTM and STM (Table 5).

**Table 5.** Comparisons of serum variables between the 2-month-old male LAP-tTA-HCV core DTM and LAP-tTA STM

Variables	DTM	STM	*p* Value
Body weight (g)	33.85 ± 3.47	32.65 ± 1.99	0.357
ALT (U/L)	88.02 ± 14.78	62.47 ± 12.98	0.001
HOMA-IR	2.195 ± 2.06	2.166 ± 2.35	0.315
TG (mg/dL)	145.5 ± 29.45	90.80 ± 6.66	<0.001
TC (mg/dL)	191.50 ± 20.46	223.50 ± 17.62	0.002
RBP4 (μg/mL)	21.1 ± 1.07	19.2 ± 2.05	0.02

LAP-tTA-HCV core, liver activator protein promoter-tetracycline transactivator-hepatitis C virus core protein; DTM, double transgenic mice; STM, single transgenic mice; ALT, alanine transaminase; HOMA-IR, homeostatic model assessment for insulin resistance; TG, triglycerides; TC, total cholesterol; RBP4, retinol-binding protein 4.

### HCV core transgenic mice hepatic IHC studies

**Figure 4.**
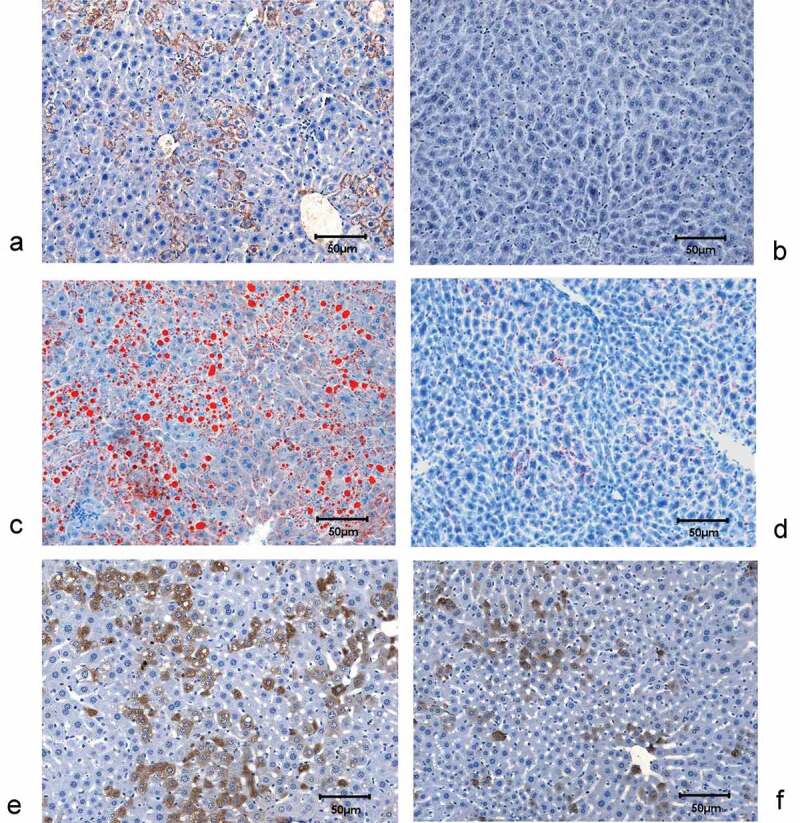
**Hepatic expression of HCV core and RBP4, and hepatic lipid accumulation of mice**. HCV core staining in the livers of LAP-tTA-HCV core DTM (a) and LAP-tTA STM (b), the HCV core protein was stained in brown. Oil red staining for the lipids in the livers of LAP-tTA-HCV core DTM (c) and LAP-tTA STM (d), the intrahepatic lipid was stained in red. RBP4 staining in the livers of LAP-tTA-HCV core DTM (e) and LAP-tTA STM (f), RBP4 was stained in brown. All figures were in 200× magnification

The LAP-tTA-HCV core DTM had high hepatic expression of HCV core (Figure 4(a)) and lipid accumulation (Figure 4(c)), by contrast, the LAP-tTA STM did not show any hepatic HCV core expression (DTM vs. STM: 26.8±13.5% vs. 0%, *p* < 0.001) (Figure 4(b)) and only exhibited minimal hepatic lipid accumulation (DTM vs. STM: 49.9±12.8% vs. 0.18± 0.06%, *p* = 0.002) (Figure 4(d)). Negligible hepatic fibrosis and inflammation were noted in both the DTM and STM livers. The DTM livers exhibited more RBP4 expression than that in the STM livers (28.39±18.66% vs. 11.57±8.55%, *p* = 0.0028), and prominent lipid vacuoles were noted in most RBP4-positive hepatocytes (Figure 4(e,f)).

## Discussion

The most compelling results of the current study are as follows: (1) Compared with controls, the CHC patients had lower baseline RBP4 levels. (2) Among CHC patients, the baseline age and levels of BMI, TG, NLR, and eGFR were associated with RBP4 levels. (3) Among SVR patients, at 24 weeks post-therapy, sex, levels of BMI, TG, uric acid, eGFR, and FIB-4 scores were associated with RBP4 levels. (4) Compared with baseline levels, SVR patients (except G3 patients) had increased 24-week post-therapy RBP4 levels, which were comparable with that of controls. (5) Hepatic expression of RBP4 levels was lower in CHC patients than the normal controls. (6) The HCV core transgenic mice (DTM) exhibited higher serum levels of TG and RBP4 than that of STM. The HOMA-IR levels were comparable between the DTM and STM. More hepatic lipid accumulation and RBP4 expression were noted in DTM than STM.

The factors consistently associated with RBP4 levels both pre-therapy in CHC patients and at 24 weeks post-therapy in SVR patients, such as BMI, TG, and eGFR, disclosed their fundamental links of RBP4, regardless of HCV infection. For example, BMI has an independent association with RBP4 levels in Asian men [30]; TG level is related to RBP4 level of participants ≥65 years [31] and is the strongest predictor of RBP4 levels in morbidly obese patients [32]; and eGFR is negatively associated with RBP4 levels [10,33]. By contrast, the pre-therapy-only factors of CHC patients such as NLR and age, and the post-therapy-only factors of SVR patients such as sex, uric acid, and FIB-4 levels suggested their potential links, direct or indirect, between HCV infection and RBP4 levels. The positive association of NLR levels and the negative association of FIB-4 scores with RBP4 levels reflected that systemic inflammation (i.e. NLR) [34] is associated with the increase, while hepatic fibrosis is associated with the decrease of RBP4 levels [35,36]. Consistently, in CHC patients, there was a significant decreasing linear trend of RBP4 dependent on both histological grading and staging progression [20], and hepatic fibrosis was associated with low RBP4 levels [11,20,37]. On the other hand, because the neutrophilic response (represented as NLR) might suppress the hepatic cytotoxic activity of hepatic T cells [34,38], the positive association of NLR with RBP4 levels actually echoes the reverse association between hepatic necroinflammatory activity/fibrosis and RBP4 levels. Moreover, hyperuricemia was inversely associated with advanced fibrosis in male CHC patients [39], it might explain, at least partly, why uric acid levels and male sex were positively associated with RBP4 levels in CHC patients. In contrast to the notion that serum RBP4 levels were lower in young compared with the elderly [40,41], the negative association of age with RBP4 levels in CHC patients might result from HCV-associated retinoid metabolism [16–18] and demands further investigation.

Compared with controls, the CHC patients had lower baseline RBP4 levels, which were reversed after viral clearance as the post-therapy RBP4 levels of the SVR patients were comparable with the controls. With only one exception [19], most studies showed lower RBP4 levels in the CHC patients than controls [11,20,37,42], even in the early disease stage [42]. Furthermore, as mentioned, hepatic inflammation and fibrosis aggravated the decrease of RBP4 levels in CHC patients [11,20,37]. The similar reversal effect of viral clearance on low RBP4 was ever seen in a Japanese G1 CHC cohort [11], and it suggested that elimination of hepatic inflammation and fibrosis subsequent to SVR in CHC patients augmented hepatic RBP4 expression. Our human IHC studies had confirmed a lower hepatic RBP4 expression in CHC patients than that in controls, and areas of inflammation or fibrosis did preclude hepatic RBP4 expression. By contrast, both the HCV-infected and mammalian expression construct encoding HCV core Huh7.5 cells showed increased RBP4 levels [43], and our HCV core transgenic mice with nonobese simple steatosis showed higher hepatic RBP4 expression and higher serum RBP4 and TG levels than the non-HCV core transgenic littermates. Given that plasma RBP4 levels correlated positively with hepatic fat [8] and serum TG levels [31,32,44], and prominent lipid vacuoles were noted in the RBP4-positive hepatocytes of the HCV core transgenic mice, it is convincible that simple steatosis with concurrent hypertriglycemia would lead to increase in RBP4 levels, regardless of HCV infection. Steatosis thus is an HCV-related, but not a HCV-specific factor for RBP4 levels. While in CHC patients, the synergistic effects of hepatic necroinflammation/fibrosis and hypotriglycemia (subsequent to HCV-related hypolipidemia [15]) in down-regulating RBP4 levels might be stronger than the sole effect of steatosis in up-regulating RBP4 levels and leads to a decrease in RBP4 levels of most patients; thus, no association could be identified between steatosis and RBP4 levels at baseline. Of note, among the SVR patients, only G3 HCV-infected patients failed to exhibit the reversal of low RBP4 levels. G3 CHC patients were believed to have viral steatosis, in contrast to metabolic steatosis in G1, G2 and G4 CHC patients [45]. As viral steatosis is related to viral load but not to metabolic factors [45], improved steatosis was usually evident in G3 but not in non-G3 patients following SVR [46]. The counterbalance between down-regulating RBP4 levels subsequent to improved steatosis and up-regulating RBP4 levels subsequent to improved hepatic necroinflammatory activity/fibrosis after SVR might diminish the pre- and post-therapy difference of RBP4 levels in G3 patients, and blunted the reversal of low RBP4 levels after SVR.

Despite the essential role of RBP4 in IR documented previously [4,5], we want to stress that the link between RBP4 and IR cannot be demonstrated in HCV infection, as the CHC cohort did not show any associations between RBP4 and HOMA-IR levels, no HOMA-IR level alteration was found in the SVR patients with the reversal of low RBP4 levels, and no differences of HOMA-IR levels were noted between the HCV core and non-HCV core transgenic littermates. Moreover, consistent with the observation that baseline RBP4 levels did not predict antiviral therapy response in CHC patients [11], the pre-therapy RBP4 levels were comparable between the SVR and non-SVR patients.

How RBP4 levels evolved had been studied in other viral infections, particularly human immunodeficiency virus (HIV), given that lipodystrophy was frequently recognized among people living with HIV receiving combination antiretroviral therapy [47]. Baseline RBP-4 levels in HIV-positive subjects were reported to be comparable with [48] or lower than those of HIV-negative controls [49]. However, RBP4 was significantly increased in HIV-infected patients with proteinuric chronic kidney disease [50], in those with overt associated lipodystrophy syndrome [51], and in those following treatment with highly active antiretroviral therapy [48]. Even in HIV/HCV-coinfected patients, levels of RBP-4 were higher for those with renal function impairment [52]. Thus, renal function and fat redistribution seemed to be crucial for RBP4 levels in the hosts, regardless of the virus kinds.

Taken together, steatosis, sex, age, uric acid, NLR, and FIB-4 levels were associated with HCV-related RBP4 levels; BMI, TG, and eGFR levels were associated with non-HCV-related RBP4 levels. The reversal of low RBP4 levels in CHC patients after SVR was not evident in G3 patients. Thus, steatosis and inflammation linked with metabolic alteration other than IR determined RBP4 levels in HCV-infected patients. These characteristic alterations and associations of RBP4 levels pave the way in probing therapeutic target for RBP4-associated cardiometabolic complications in CHC patients after viral clearance.
